# Investigations on the optical forces from three mainstream optical resonances in all-dielectric nanostructure arrays

**DOI:** 10.3762/bjnano.14.53

**Published:** 2023-06-02

**Authors:** Guangdong Wang, Zhanghua Han

**Affiliations:** 1 Shandong Provincial Key Laboratory of Optics and Photonic Devices, Center of Light Manipulation and Applications, School of Physics and Electronics, Shandong Normal University, Jinan 250358, China

**Keywords:** all-dielectric nanostructures, anapole, optical force, quasi-bound states in the continuum, toroidal dipole

## Abstract

Light can exert radiation pressure on any object it encounters, and the resulting optical force can be used to manipulate particles at the micro- or nanoscale. In this work, we present a detailed comparison through numerical simulations of the optical forces that can be exerted on polystyrene spheres of the same diameter. The spheres are placed within the confined fields of three optical resonances supported by all-dielectric nanostructure arrays, including toroidal dipole (TD), anapoles, and quasi-bound states in continuum (quasi-BIC) resonances. By elaborately designing the geometry of a slotted-disk array, three different resonances can be supported, which are verified by the multipole decomposition analysis of the scattering power spectrum. Our numerical results show that the quasi-BIC resonance can produce a larger optical gradient force, which is about three orders of magnitude higher than those generated from the other two resonances. The large contrast in the optical forces generated with these resonances is attributed to a higher electromagnetic field enhancement provided by the quasi-BIC. These results suggest that the quasi-BIC resonance is preferred when one employs all-dielectric nanostructure arrays for the trapping and manipulation of nanoparticles by optical forces. It is important to use low-power lasers to achieve efficient trapping and avoid any harmful heating effects.

## Introduction

Optical forces have two components (i.e., scattering force and gradient force [1]) and the motion of tiny particles within an optical field is determined by both of them. The scattering force originates from the photon momentum transferred to the particle caused by the scattering and absorption of photons. As a result, the scattering force is along the direction of light propagation, which is not conducive for object capturing. The gradient force is along the gradient direction of the non-uniform distribution of the light intensity in space, and is well-known for its application in optical tweezers [2]. Therein a strong laser beam generates a piconewton level of force, which can be used to manipulate small dielectric particles, including biological entities such as DNA, enzymes, and cells. The underlying physics of nanoparticle manipulation by optical tweezers can be interpreted as the trend of the particle to move to a region of high field strength to reduce its energy [3]. Unfortunately, due to the diffraction limit, light cannot be focused onto the subwavelength volume; so it is very difficult for optical tweezers to capture nanoscale objects. Recently, plasmonic nanotweezers have proved their capability to effectively capture subwavelength nanoparticles by overcoming the diffraction limit [4], which has aroused broad research interest. However, due to the high loss of metals, the Joule heating effect caused by the absorption of light leads to increasing temperatures of plasmonic nanotweezers, and may generate a detrimental effect to the captured particles. In this context, all-dielectric nanostructures made from lossless materials are preferable to be used. With the possibility to support various types of electromagnetic resonances (e.g., toroidal dipole (TD), anapole, or bound state in the continuum (BIC)) which are current in focus nanophotonics research topics, all-dielectric nanostructures have proved themselves to be a good platform for light–matter interactions and an advantageous alternative to their plasmonic counterparts.

A TD resonance is produced by the flow of electric currents on the surface of a torus along its meridians, which excite a set of magnetic dipoles (MDs) arranged head-to-tail into a closed loop [5]. First proposed by Zel'dovich in atomic physics [6], and existing widely in elementary particles and condensed matter, such as multiferroic materials [7], the TD cannot interact directly with electromagnetic waves and is often masked by electric dipoles (EDs) or magnetic dipoles (MDs) with stronger responses. However, the TD has a unique current distribution, which can generate a strong near-field localization effect, so it has broad application scenarios [8]. The anapole [9] mode is produced by nanostructures with both ED and TD excitations at the same time. The ED and TD moments generate radiation fields of the same pattern but with opposite phases, leading to the destructive interference of radiation fields. In the far-field region, it can be observed that the radiation field significantly decreases or even vanishes in the scattering spectrum at a specific wavelength. In dielectric materials, the anapole mode is mainly confined to the interior of the structure and does not strongly extend into the surrounding medium [10].

The BIC is a wave excitation which remains spatially localized while the frequency co-exists within a continuum of radiations. Although this concept was first proposed in the field of quantum mechanics [11], it has also attracted much attention in recent years in photonics [12] due to its ability to achieve high-quality (*Q*) factor resonance and the associated high-field enhancement. The ideal BIC has an infinite *Q*-factor and zero resonance linewidth, so it can only exist as a mathematical quantity and cannot be excited by free-space radiations. However, with some intentional perturbations in the geometry or objective loss channels, such as surface roughness, a BIC will turn into a quasi-BIC mode with both the *Q*-factor and resonant bandwidth becoming limited. Many applications of quasi-BICs have been reported, including ultrasensitive sensing [13], ultra-narrow bandwidth filters [14], and enhanced nonlinear effects [15]. The BICs are usually categorized into several types [16], and in this work we are concerned with the symmetry-protected BIC. This type of BIC is formed due to a symmetry mismatch between the mode distribution and the free space radiations (e.g., a plane wave). When a structural perturbation is introduced into the system to break the symmetry, the coupling to external radiations can be enabled with the efficiency controlled by the level of perturbation. These quasi-BICs can provide higher *Q*-factors and usually higher field enhancement than those of the TD resonance and the anapole mode. As a result, the quasi-BICs are expected to provide a larger field gradient which provides higher capability in the applications of nanoparticle capturing. However, a complete investigation and comparison of the optical forces which can be provided by these resonances is still missing.

In this work, we present some numerical results to compare the optical trapping capability provided by all-dielectric nanostructures based on the excitation of these three different modes. Using an array of high-index silicon disks with elaborately designed slots, all these three resonances can be supported by the same platform. The scattering spectra of these modes are analyzed by the multipole decomposition method, which ensures that strong TD response, the anapole mode composed of simultaneously working TD and ED momentums, and the quasi-BIC resonance arising from an out-of-plane MD mode can all be excited using similar structures. The transmission spectrum through a periodic disk array and the electromagnetic fields in resonance were numerically investigated by the finite element method (FEM) implemented in the commercial software COMSOL Multiphysics. In all calculations, we investigated the generated optical forces on nanoscale polystyrene (PS) spheres in the slot of the all-dielectric nanostructures. All these spheres have their own response to the incident radiations (e.g., Mie resonances). However, we noted that due to the small size of the spheres and the relatively lower refractive index of the polymeric material, these resonances by the spheres are beyond the spectrum of our interest. The presence of PS spheres only leads to some spectral shift of the resonances supported by the silicon nanostructure, which have been fully considered. Using the Maxwell stress tensor (MST) technique [17], the generated optical forces on these spheres placed within the near field of these modes are calculated, analyzed, and compared. We found that the optical force applied on the same PS sphere by the quasi-BIC mode under the same excitation power is about three orders of magnitude larger than those from the other two modes. Furthermore, our calculations show that even for nanoscale spheres, the quasi-BIC resonance can still provide a large optical force that allows for an effective trapping of these spheres.

## Results and Discussion

**Figure 1:**
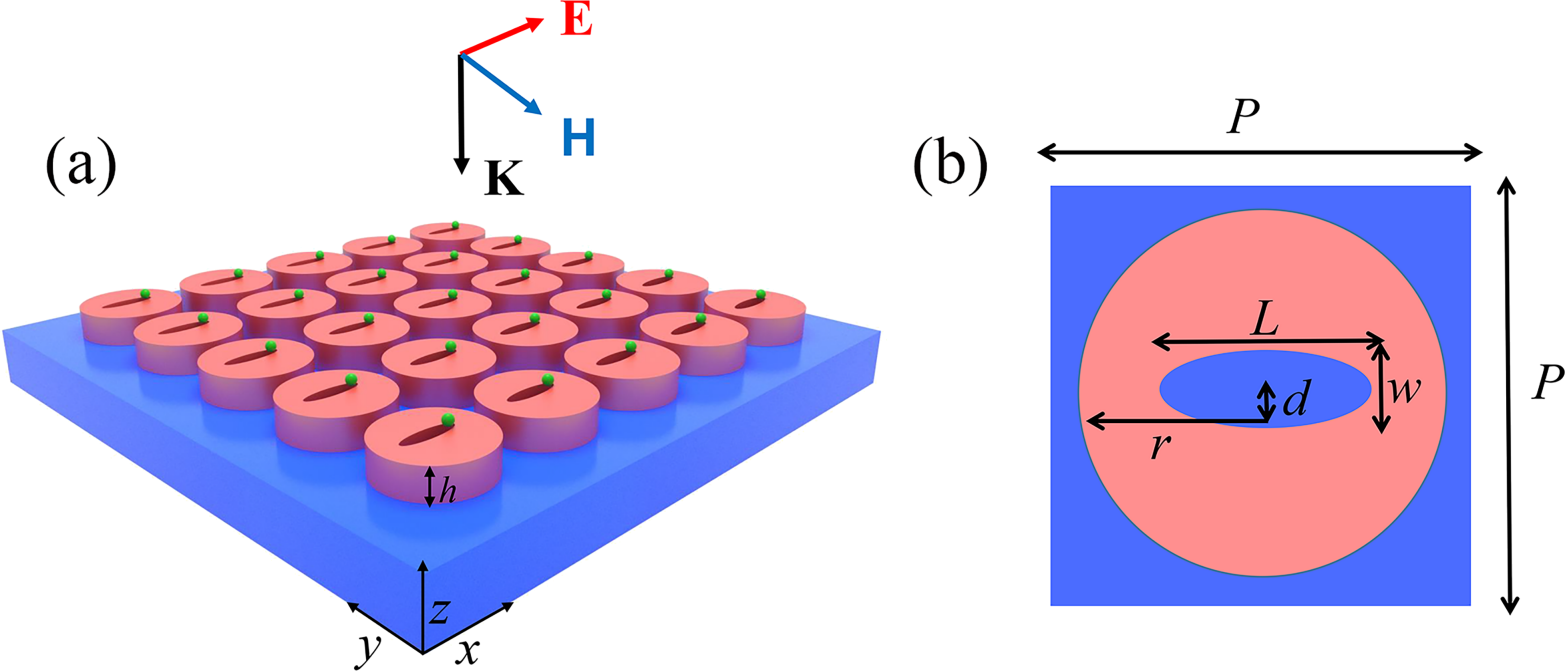
(a) Schematic overview of the metasurface structure composed of a silicon slotted-disk array on a quartz substrate. The trapped nanoscale particles and the incident plane wave are also illustrated. (b) Top view of one unit cell of the slotted-disk array. The geometrical parameters are as follows: array period *P* = 880 nm, disk radius *r* = 390 nm, thickness *h* = 225 nm, slot length *L* = 420 nm, and slot width *w* = 90 nm. In this work, all these parameters remain constant while the number of slots and the distance *d* between the centers of the disk and the slot depend on the specific resonance to be excited, which is discussed in the main text.

Figure 1a illustrates the schematic of the metasurface structure, where the small green objects on top of the silicon disks represent the trapped PS spheres. The metasurface consists of a 225 nm thick array of silicon disks on a quartz substrate with elliptical slots etched perforating each disk. The number and positions of the slots depend on the specific mode to be excited, and may be different for those three kinds of resonances. Figure 1b presents the top view of one unit cell, where the geometrical parameters are described in the caption. A linearly polarized plane wave is normally incident to the metasurface, with the polarization along the *x*-direction to excite all the TDs, anapoles, and the quasi-BIC resonances. For all cases, the metasurface structure is assumed to be immerged in water (*n* = 1.31) to model a realistic suspending condition for the PS nanospheres. The optical forces on the PS at the excitation values of those three resonances are all calculated based on the MST technique, with the input power intensity set as 1 mW/µm^2^.

Within the framework of classical electrodynamics, the components of the total time-averaged force *F* acting on an illuminated object can be calculated using the surface integral:


[1]

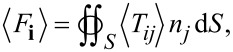



where *S* is a closed surrounding surface, *n* is the unit vector perpendicular to and pointing toward the outside of the surface, and ⟨*T_ij_*⟩ is the time-averaged MST [18] defined by


[2]

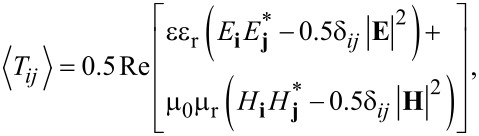



where the indices *i* and *j* denote *x*, *y*, or *z* components of the electric or magnetic field; ε_r_ and μ_r_ are the relative permittivity and the relative permeability of the surrounding medium, respectively. In this work, we use a small virtual cube to accommodate a small object (i.e., the PS sphere) and all surfaces of the cube are in water. The electromagnetic fields at the six surfaces of the virtual cube are used to calculate the optical force according to Equation 2. With numerical calculations, the local electromagnetic fields within the structure at the excitation value of a specific resonance can be calculated. From this value one can estimate the generated optical forces combining Equation 1 and Equation 2.

### Optical force with toroidal dipole excitation

**Figure 2:**
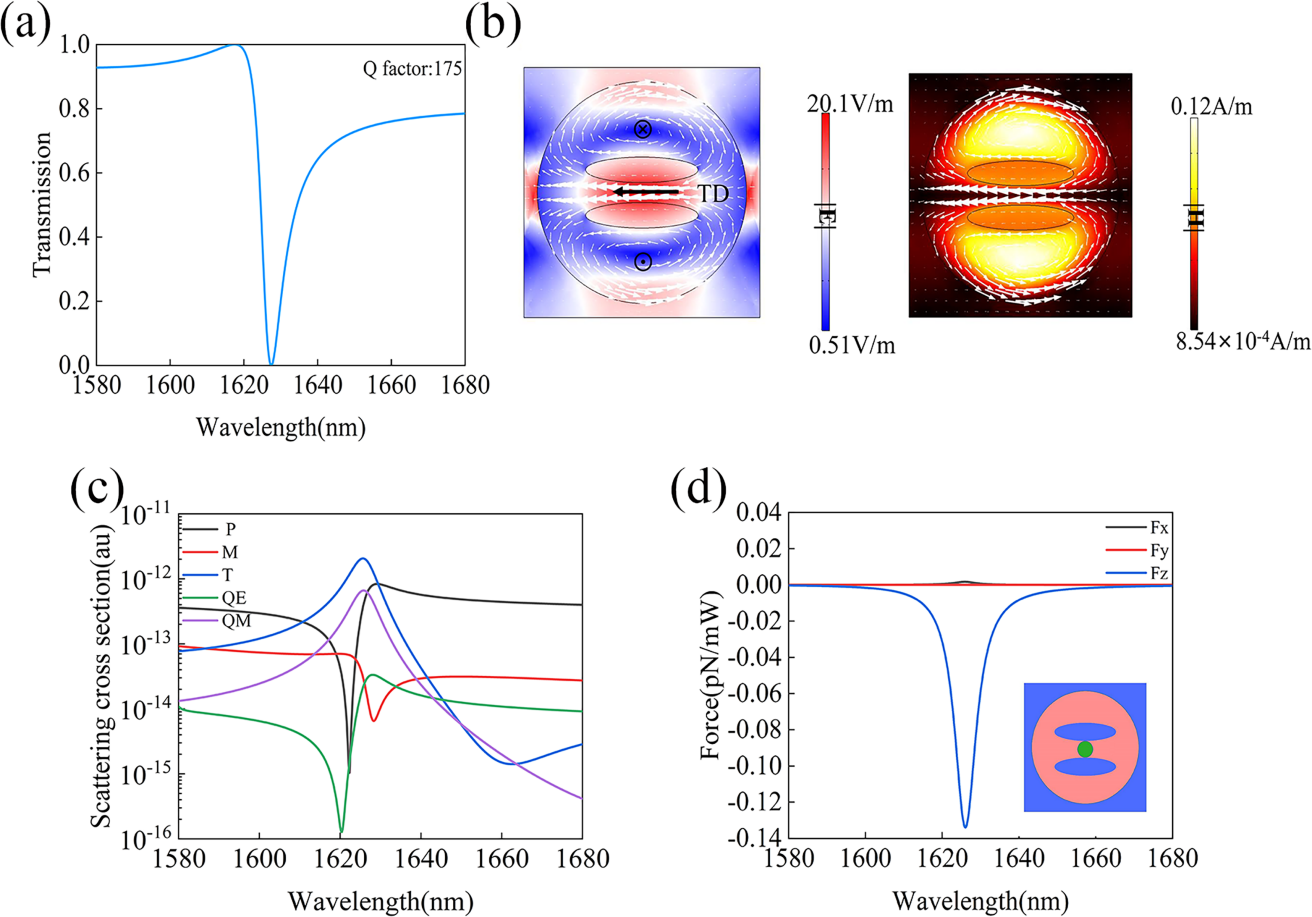
(a) Transmission spectrum through the metasurface supporting the TD resonance. (b) Electric and magnetic field distributions at the TD resonance, where the white arrows represent the displacement current vectors. (c) Multipole analysis of the overall scattering spectrum. (d) The calculated optical force exerted on the PS sphere as a function of the wavelength; the inset shows the geometry of one unit cell of the double-slotted silicon disk array. Both slots are shifted by a distance of *d* = 80 nm from the center of the disk.

**Figure 3:**
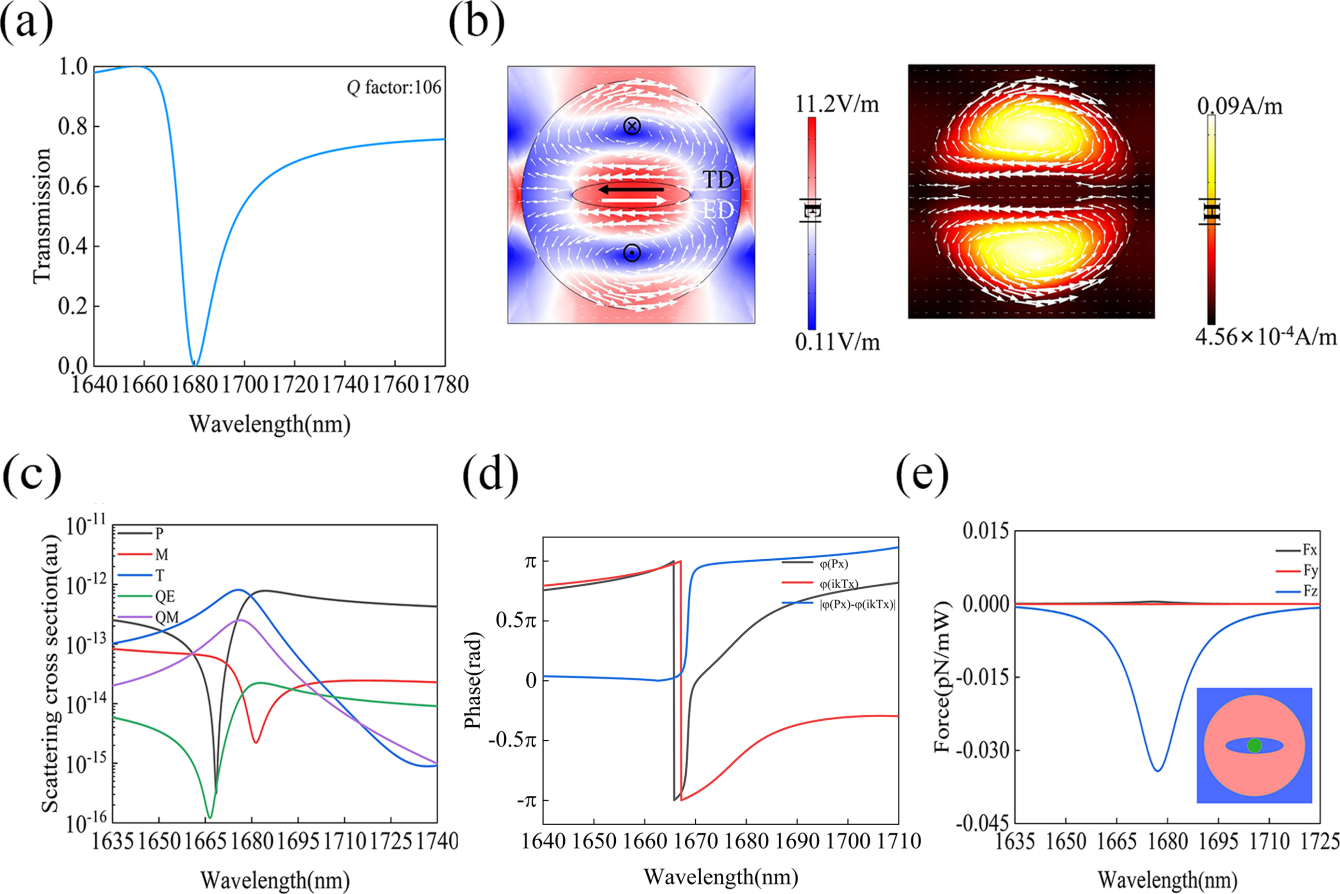
(a) Transmission spectrum through the metasurface supporting the anapole resonance. (b) Electric and magnetic field distributions at the anapole wavelength, where the white arrow represents the displacement current vectors. (c) Multipole analysis of the overall scattering spectrum. (d) The phases of *P*, *ikT*, and their phase difference. (e) The optical force on the PS sphere as a function of wavelength; the inset shows one unit cell of the geometry and the position of the PS sphere.

**Figure 4:**
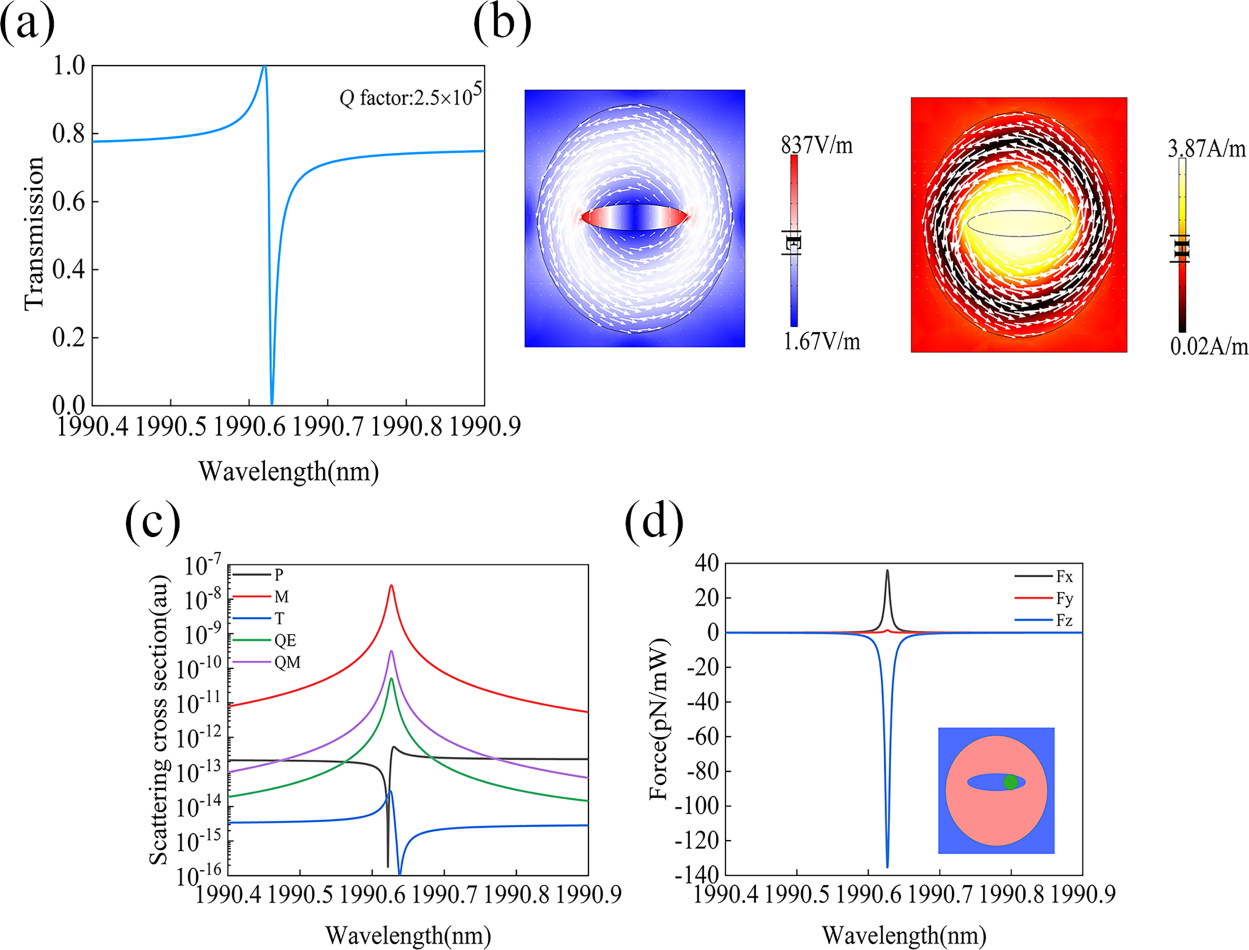
(a) Calculated transmission spectrum through the metasurface supporting the quasi-BIC resonance. (b) Electric and magnetic field distributions at the quasi-BIC resonance, where the white arrows represents the displacement current vectors. (c) Contributions from different multipoles to the overall scattering as a function of wavelength. (d) Calculated optical force spectrum on the PS sphere; the inset shows one unit cell of the geometry and the position of the PS.

It is known that the excitation of the TD resonance requires the presence of a set of magnetic dipoles arranged head-to-tail to form a closed loop. This kind of TD resonance is usually called magnetic TD. In contrast, the TD response due to a circular configuration of electric dipoles is called electric TD. In this work, we focus only on the magnetic TD and then use only TD instead for simplicity. To fulfill the requirement of using a closed loop of magnetic dipoles, two perforating elliptical slots are used with mirror symmetry with respect to the *x*–*z* plane across the center of the disk, and each slot is shifted from the disk center by a distance of *d* = 80 nm. Figure 2a presents the calculated transmission spectrum through this structure with the incident electric field polarized along the *x*-direction. It can be seen that a strong asymmetric optical resonance of the Fano-type is located around 1627 nm with a resonance *Q*-factor of 175. The electric and magnetic field amplitude distributions at the resonance wavelength are plotted in Figure 2b, where the white arrows represent the electric displacement current vectors. It is seen that the electromagnetic field is well confined within the silicon disk. Although the optical force is dependent on the laser power, we should note that the light transmission spectra through the array is not if the nonlinear effect is ignored. So in the calculations of the transmission spectra and the on-resonance field distributions, the electric field magnitude of the incident plane wave is set as 1 [V/m]. Then the maximum electric field in Figures 2b, 3b, and 4b straightforwardly give the enhancement capability of the corresponding resonance. Moreover, two circular displacement currents with reverse rotational directions are formed in the *x*–*y* plane of the disk, indicating that two MD resonances with opposite momentum directions are formed. Since the whole structure is symmetric in the *y*-direction, the two MDs have the same amplitude. These two MDs form a closed loop, leading to a TD momentum along the *x*-axis (see the left part of Figure 2b). In other words, a plane wave polarized along the *x*-axis can excite a TD resonance in this structure. In order to further confirm this, we analyzed the contributions to the total scattering power spectrum from five different multipoles, including the ED (P), MD (M), electric quadrupole (QE), magnetic quadrupole (QM), and magnetic toroidal dipole (T) in the Cartesian coordinate system using the multipole decomposition technique. The multipole expansion is achieved based on the electric displacement current using the following formula:


[3]

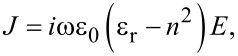



where ε_0_ represents the vacuum dielectric constant, *n* is the refractive index of the uniform background above the metasurface structure, in which the virtual domain used to calculate the optical force is defined and the displacement currents used for multipole analysis are obtained, and *E* is the total electric field inside the disk. The multipole analysis is normally used for isolated nanoparticles. However, for periodic nanostructures, especially when the unit cell is much smaller than the incident wavelength and all dipoles can be assumed to oscillate in phase, the formula of the multipole decomposition can also be used [19–20]. The obtained results will provide important insight into the origin of the observed resonances in the transmission/reflection spectra of the periodic structures. Following this approach, we performed the multipole analysis and the results are presented in Figure 2c. It is evident that at the resonance wavelength of 1627 nm, the TD contribution in the scattering power dominates, while the ED and MD contributions are both suppressed. The suppressing is due to the excitation of multiple EDs and MDs in the structure, which will cancel each other out in the far-field radiations. For example, the displacement current distribution vector diagram on the left side of Figure 2b shows that the strong displacement current excited between the two slots will be balanced by the two external displacement currents on the top and bottom sides of the disc (i.e., the ED is weaker in this case). At the same time, we can also see from Figure 2c that the higher-order QE resonance is significantly suppressed, while the QM resonance is slightly lower than the TD resonance. These results confirm that the Fano-type transmission dip in Figure 2a can be considered as resulting from the coupling between the excited TD and QM resonances.

Because the optical gradient force is proportional to the gradient of the local electric field intensity (i.e., *F*_grad_ ∝ ∇|*E*(*r*)|^2^), the PS sphere gets trapped where the electric field has the highest value. In the metasurface structure supporting the TD resonance, the PS sphere is eventually stabilized between the two elliptical slots. Figure 2d shows the calculated optical force on the PS sphere using Equation 1 as a function of the wavelength. The PS sphere has a diameter of 50 nm and is assumed to be located at the position of (*x*_0_, *y*_0_, *z*_0_) = (0, 0, 260 nm), which is 35 nm above the top surface of the disk, as illustrated by the inset of Figure 2d. It can be seen from the results that the peak of the optical force is achieved at the TD resonance, with the component of *F_z_* being much higher than the other two. The peak value of *F_x_* on the PS sphere is about 0.0017 pN/mW, *F_y_* = 0, and *F_z_* about −0.134 pN/mW. The counterintuitive nonzero *F_x_* value even when the structure is symmetric along the *x*-direction is associated with the slightly asymmetric field distributions at the peak of a Fano resonance [21], which can be seen in Figure 1b. This indicates that the PS sphere does not get stably trapped at a fixed position above the disk surface, but is pulled towards the center of the disk in the negative *z*-direction.

### Optical force with the anapole excitation

The anapole resonance needs the simultaneous excitations of a TD and an ED, whose radiations into the far field should be out of phase to form a destructive interference to eliminate the overall scattering. We used one elliptical slot at the center of the silicon disk (i.e., *d* = 0 nm) while the other geometrical parameters remain the same as in Figure 1. Figure 3a presents the simulated transmission spectrum through this metasurface structure with the incident electric field along the *x*-direction. One can see that a strong optical resonance with similar asymmetric Fano profile as shown in Figure 2a is located around 1680 nm, with a *Q*-factor of about 106. The results of the multipole analysis to be discussed later confirm the anapole characteristic of this resonance. The electric and magnetic field distributions at the resonance are shown in Figure 3b, where the white arrows also represent the electric displacement current vectors. The enhancement of the electric and magnetic fields at the resonance is smaller compared to those at the TD resonance in Figure 2b. As can be seen from Figure 3b, two current loops with opposite circulation directions are excited on different sides of the elliptical slot, indicating that the TD momentum is along the *x*-axis. Although similar current loops can be observed in the lower and upper semicircles of the silicon disk in both Figure 2b and Figure 3b, we note that a much stronger current at the upper and lower edges of the silicon disk can be found in Figure 3b, suggesting that a nonzero net ED remains in this metasurface. It is the destructive interference between the TD and the net ED resonances which leads to the excitation of the final anapole mode.

To further analyze the contributions from different multipoles to the observed resonance in Figure 3a, we performed a similar multipole analysis of the scattered power as a function of wavelength. It is clearly seen in Figure 3c that the TD and ED are the two major components in this system. At the resonance wavelength of 1680 nm, the ED and TD have the same magnitude. In addition, we can clearly see in Figure 3d that TD and ED have opposite phases and the phase difference between TD and ED at 1680 nm |(*P*)−(*ikT*)| is approximately equal to π. These results confirm that the resonance in Figure 3a is mainly caused by the destructive interference between ED and TD moments, which in turn leads to the anapole response. Figure 3e shows the calculated optical force as a function of wavelength on a PS sphere with a diameter of 50 nm. The position of the PS sphere with respect to the geometry of the anapole system is illustrated by the inset of Figure 3e. The PS sphere is assumed to be at the same position as that in Figure 2 (i.e., (*x*_0_, *y*_0_, *z*_0_) = (0, 0, 260 nm)). It can be seen that the major optical force component is still *F_z_*, which is consistent with the results obtained with the TD resonance presented in Figure 2d. However, both peak values of *F_x_* and *F_z_* achieved with the anapole resonance are slightly smaller than those with the TD resonance. For *F_z_*, the force relative to the TD resonance is −0.134 pN/mW, which is 3.92 times larger than that from the anapole. For *F_x_*, the contrast is about 3.4 times, 0.0017 pN/mW with the TD versus 0.0005 pN/mW with the anapole. The smaller optical force achieved with the anapole compared to that of the TD resonance is attributed to a weaker local field enhancement achieved at the anapole resonance. From Figure 3a and Figure 3b, one can see a smaller *Q*-factor (106) at the anapole resonance and a weaker local electric field enhancement (11.2) compared to those of the TD resonance.

### Optical force with the quasi-BIC excitation

The quasi-BIC mode with unprecedented ultra-high *Q*-factor and associated ultra-high field enhancement is expected to provide a large optical force, considering that the optical gradient force is directly proportional to the field enhancement. To have a fair comparison, we used the same silicon disk array which supports an out-of-plane MD resonance. This resonance can not be excited by an incident plane wave due to the symmetry incompatibility, giving rise to a BIC resonance of the symmetry-protected type. In order to excite the resonance, we intentionally shifted the elliptical slot in the metasurface structure supporting the anapole by *d* = 5 nm along the *y* direction with respect to the disk center. Then the symmetry of the whole system was slightly broken, leading to the transition of the BIC into a quasi-BIC resonance, which has the possibility of being excited by a plane wave. Figure 4a presents the simulated transmission spectrum through this metasurface structure under the excitation of an *x*-polarized plane wave. A strong and sharp optical resonance with also an asymmetric Fano profile is found located near 1990.63 nm with a *Q*-factor of about 2.5 × 10^5^. Figure 4b presents the electric and magnetic field distributions at the resonance, where the white arrows still represent the electric displacement current vectors. The field enhancement of the quasi-BIC system is further enlarged by approximately one order of magnitude compared to those achieved at both TD and anapole resonances. Figure 4b also shows that the maximum field enhancement occurs at both ends of the elliptical slot, where the particles are more easily captured. One can also see from Figure 4b that a counterclockwise loop of the electric displacement current is excited inside the silicon disk, which is related with an MD moment along the *z*-axis.

To further investigate the origin of the supported quasi-BIC resonance, we calculated the multipole contributions to the overall scattered power at different wavelengths. From the results in Figure 4c, it is clear that the scattered power from the MD resonance dominates the resonance at the wavelength of 1990.63 nm (i.e., the quasi-BIC response is caused by the MD resonance). Under the *x*-polarized incident light excitation, the dielectric structure generates a displacement current which is roughly, although not exactly, centrosymmetric. So the electric current in one quadrant of the disk will cancel that in the opposite quadrant, leading to the smallest ED moment among all the multipoles. The PS sphere has a diameter of 50 nm and is assumed to be located at the position (*x*_0_, *y*_0_, *z*_0_) = (150 nm, 5 nm, 260 nm), as illustrated by the inset of Figure 4d (i.e., 35 nm above the top surface of the disk). The geometry of the metasurface unit cell and the position of the PS sphere are shown in the inset of Figure 4d. Figure 4d presents the optical force spectrum, which shows that the optical force on the PS sphere at the wavelength of the quasi-BIC is much higher (*F_x_* = 36 pN/mW, *F_y_* = 1.4 pN/mW, *F_z_* = −135 pN/mW) compared to those achieved with the TD resonance and the anapole. This higher optical force is due to a higher *Q*-factor (2.5 × 10^5^) and the associated much stronger electric field enhancement (837 times) with the quasi-BIC mode. The results show that even at a relatively low laser power intensity, the PS sphere located in the metasurface supporting the quasi-BIC resonance will be subjected to a strong optical force, which means the PS sphere can be stably captured.

## Discussion and Conclusion

Table 1 presents a direct comparison of different characteristics at the excitation of three resonances investigated in this work. It can be seen that at the same excitation power intensity, the optical force generated by the quasi-BIC resonance is about three orders of magnitude larger than those generated by the TD and anapole resonances. The contrast in the achieved optical forces is consistent with the difference in the resonance *Q*-factor, which is intrinsically connected with the local electromagnetic field enhancement. We should note that all these values in Table 1 are rough numbers with no optimization (e.g., the *Q*-factor of the quasi-BIC resonance can be controlled by the level of symmetry breaking (the size and position of the elliptical slot)). However, the results in Table 1 with different orders of magnitude in the achieved optical forces still provide a straightforward contrast regarding the characteristics of optical forces with different kinds of resonances supported by all-dielectric nanostructures.

**Table 1:** Comparison of different characteristics with the three resonances studied in this work.

Resonance	*Q*-factor	*E* field enhancement	*F_x_* (pN/mW)	*F_y_* (pN/mW)	*F_z_* (pN/mW)

TD	175	20.1	0.0017	0	−0.134
anapole	106	11.2	0.0005	0	−0.0342
quasi-BIC	2.5 × 10^5^	837	36	1.4	−135

We should note that in the above results summarized in Table 1, the *Q*-factor of the quasi-BIC is overwhelmingly larger than the other two types of resonances. However, the ultra-high *Q*-factor of the quasi-BIC mode results from a collective behavior of all array elements. When only a single nanostructure is used the *Q*-factor can be significantly reduced, although the quasi-BIC mode can also be supported either due to symmetry incompatibility [22] or due to the strong coupling between different modes [23]. In general, to trap multiple nanoparticles using periodic nanostructures the quasi-BIC resonance is usually the first choice. However, to trap a single particle with isolated nanostructures, further investigation needs to be performed for a better comparison.

In conclusion, to the best of our knowledge, we systematically investigated for the first time and compared the optical trapping capability of three mainstream optical resonances that can be supported by all-dielectric nanostructure arrays. The high *Q*-factor and strong electric field enhancement that can be provided by the quasi-BIC resonance makes it a more suitable option over other resonances for the trapping of multiple nanoparticles using periodic structures. These results will provide useful guidelines when one designs nano-optical tweezers system to capture nanoscale particles. The low absorption losses, high laser power damage threshold as well as the large optical force that can be achieved with lossless all-dielectric nanostructures provide a better choice compared to the metallic counterpart in optical capturing of nanoscale particles and may have broad applications in medicine and biology.
